# Supplementary material for: Muscle Spatial Transcriptomic Reveals Heterogeneous Profiles in Juvenile Dermatomyositis and Persistence of Abnormal Signature After Remission
Source: Cells. 2025 Jun 19;14(12):939. doi: 10.3390/cells14120939 (PMC12190613; doi:10.3390/cells14120939)
Supplement: Supplementary file 1 [file cells-14-00939-s001.zip › cells-3655567-supplementary.pdf]

Tragin et al-Supplementary file

Methods

Histology, immunohistochemistry, morphometric analyses, association between spatial transcriptomic results and myofibre alterations.

7 µm cryosections of muscle samples (CryoStar NX70, Thermo Fisher Scientific, Waltham, MA, USA) were dried for 30 minutes at room temperature and stored at -80°C before staining. For immunofluorescence staining, sections were thawed for 30 minutes at room temperature and then hydrated with 1X PBS for 10 minutes. They were permeabilized in Triton 0.5% for 5 minutes and then, incubated in 10% BSA (Sigma-Aldrich) for 30 minutes. Sections were incubated with primary antibodies diluted in 1X PBS overnight at 4°C: anti-Laminin rabbit polyclonal antibody (Cat # L9393, 1:500; Sigma-Aldrich) or anti-collagen VI Mouse monoclonal antibody (Cat # MAB1944, 1:1000; Chemicon, Sigma-Aldrich). Then, sections were incubated with appropriate conjugated secondary antibodies for 30 minutes at 37°C and mounted with Fluoromount-G Mounting Medium (Invitrogen). Pictures were acquired using a fluorescent microscope (ZEISS Axio Imager D1). For double immunofluorescence stainings, sections were hydrated with 1X PBS for 10 minutes and then were fixed in 4% PFA for 10 minutes. After this step, the same protocol was applied as above. Sections were incubated with Anti-Laminin rabbit polyclonal antibody (Cat # L9393, 1:500; Sigma-Aldrich) and anti-CD31 endothelial cell Monoclonal Mouse antibody (Cat # M0823, 1:50; Dako, Agilent). Morphometric analysis were performed using a macro developed on Fiji, an open access image processing package based on ImageJ®. This macro allowed to detect and quantify the number of myofibres in sections and to obtain the area or minor diameter values. The other macro used, detected the membrane and vessels associated to each myofibre. To evaluate fibrosis, Fiji was used to detect the whole section and then measure the Collagen VI area fraction.

Myofibre alterations in JDM muscles were classified as follows: (i) Perifascicular atrophy characterized by the elective atrophy of marginal peripheral myofibre layers of myofibres (perifascicular atrophy); (ii) ischaemic changes composed of punch-out vacuoles and microinfarcts consisting of small foci of contiguous necrotic or regenerating fibres; (iii) overall myofibre atrophy; and (iv) myopathic changes with rounded appearance of myofibres and internalized nuclei. From this classification, we delineated on JDM muscle biopsies areas characterized by the prominence of one of the patterns. Then, blinded to the Visium spatial transcriptomic data, these areas were superimposed on Visium spots using 10X Genomics software Loupe Browser 7.0.1. Each Visium spot was assigned to the molecular signature showing the maximal percentage after deconvolution. Distribution

of maximal signatures for each histopathological annotation were calculated, and contingency tables were statistically compared using Fisher test from Stats v.4.4.2 R package.

Supplemental Table S1 List of gene markers for each of 5 signatures. Reference-free deconvolution provided an expression matrix of genes in each signature. For each signature, marker genes were extracted based on gene expression levels and a fold change was calculated as described in Miller *et al.* (2022).

Ensemble identifier	Gene marker	log2FC	Molecular Signature
ENSG00000198899	ATP6	3,20	1
ENSG00000228253	ATP8	3,02	1
ENSG00000198786	ND5	2,47	1
ENSG00000198840	ND3	2,42	1
ENSG00000004799	PDK4	2,24	1
ENSG00000198804	COX1	2,11	1
ENSG00000198938	COX3	1,54	1
ENSG00000198727	CYTB	1,36	1
ENSG00000212907	ND4L	1,24	1
ENSG00000112306	RPS12	-1,46	1
ENSG00000184076	UQCR10	-1,64	1
ENSG00000170290	SLN	-1,77	1
ENSG00000101608	MYL12A	-2,21	1
ENSG00000130595	TNNT3	-2,29	1
ENSG00000129170	CSRP3	-2,30	1
ENSG00000106211	HSPB1	-2,37	1
ENSG00000168530	MYL1	-2,38	1
ENSG00000101470	TNNC2	-2,43	1
ENSG00000128591	FLNC	-2,60	1
ENSG00000154553	PDLIM3	-2,64	1
ENSG00000075624	ACTB	-2,69	1
ENSG00000130598	TNNI2	-3,19	1
ENSG00000140416	TPM1	-3,48	1
ENSG00000156508	EEF1A1	-3,51	1
ENSG00000166710	B2M	-3,66	1
ENSG00000180209	MYL11	-3,84	1
ENSG00000125414	MYH2	-4,02	1
ENSG00000244734	HBB	27,11	2
ENSG00000178878	APOLD1	4,39	2
ENSG00000198886	ND4	2,69	2
ENSG00000212907	ND4L	2,52	2

ENSG00000198712	COX2	2,09	2
ENSG00000198727	CYTB	2,02	2
ENSG00000182676	PPP1R27	1,60	2
ENSG00000185559	DLK1	1,52	2
ENSG00000117479	SLC19A2	1,40	2
ENSG00000198938	COX3	1,11	2
ENSG00000004799	PDK4	1,09	2
ENSG00000142089	IFITM3	-1,09	2
ENSG00000198899	ATP6	-1,39	2
ENSG00000142156	COL6A1	-1,45	2
ENSG00000173641	HSPB7	-1,46	2
ENSG00000101439	CST3	-1,62	2
ENSG00000204287	HLA-DRA	-1,68	2
ENSG00000106211	HSPB1	-1,93	2
ENSG00000101470	TNNC2	-1,94	2
ENSG00000206503	HLA-A	-2,27	2
ENSG00000011465	DCN	-2,37	2
ENSG00000180209	MYL11	-2,47	2
ENSG00000168530	MYL1	-2,58	2
ENSG00000154553	PDLIM3	-2,61	2
ENSG00000204525	HLA-C	-2,76	2
ENSG00000125414	MYH2	-2,84	2
ENSG00000130595	TNNT3	-2,95	2
ENSG00000166710	B2M	-2,98	2
ENSG00000170290	SLN	-3,58	2
ENSG00000148677	ANKRD1	8,01	3
ENSG00000159251	ACTC1	5,05	3
ENSG00000133020	MYH8	4,87	3
ENSG00000109063	MYH3	4,14	3
ENSG00000138435	CHRNA1	3,91	3
ENSG00000165887	ANKRD2	2,95	3
ENSG00000101608	MYL12A	2,83	3
ENSG00000150779	TIMM8B	2,63	3
ENSG00000170290	SLN	2,56	3
ENSG00000180209	MYL11	2,53	3
ENSG00000086967	MYBPC2	2,49	3
ENSG00000106992	AK1	2,43	3
ENSG00000168530	MYL1	2,31	3
ENSG00000272573	MUSTN1	2,21	3
ENSG00000166025	AMOTL1	2,10	3
ENSG00000118729	CASQ2	1,91	3
ENSG00000140416	TPM1	1,84	3

ENSG00000106211	HSPB1	1,82	3
ENSG00000133110	POSTN	1,71	3
ENSG00000267855	NDUFA7	1,68	3
ENSG00000126709	IFI6	1,65	3
ENSG00000109061	MYH1	1,62	3
ENSG00000154553	PDLIM3	1,35	3
ENSG00000101470	TNNC2	1,26	3
ENSG00000128591	FLNC	1,16	3
ENSG00000130595	TNNT3	1,05	3
ENSG00000125414	MYH2	1,02	3
ENSG00000101439	CST3	-1,07	3
ENSG00000163430	FSTL1	-1,18	3
ENSG00000142156	COL6A1	-1,27	3
ENSG00000008517	IL32	-1,31	3
ENSG00000111341	MGP	-1,59	3
ENSG00000198899	ATP6	-1,59	3
ENSG00000198786	ND5	-1,60	3
ENSG00000198712	COX2	-1,74	3
ENSG00000198804	COX1	-1,79	3
ENSG00000026025	VIM	-1,82	3
ENSG00000163453	IGFBP7	-1,82	3
ENSG00000198840	ND3	-2,14	3
ENSG00000011465	DCN	-2,26	3
ENSG00000148180	GSN	-2,26	3
ENSG00000142089	IFITM3	-2,49	3
ENSG00000198938	COX3	-3,13	3
ENSG00000198727	CYTB	-3,68	3
ENSG00000198886	ND4	-5,33	3
ENSG00000211592	IGKC	4,90	4
ENSG00000173369	C1QB	4,71	4
ENSG00000107796	ACTA2	4,10	4
ENSG00000187608	ISG15	4,06	4
ENSG00000100600	LGMN	3,79	4
ENSG00000185885	IFITM1	3,61	4
ENSG00000115415	STAT1	3,57	4
ENSG00000119917	IFIT3	3,46	4
ENSG00000182326	C1S	3,42	4
ENSG00000111341	MGP	3,40	4
ENSG00000185745	IFIT1	3,36	4
ENSG00000019582	CD74	3,18	4
ENSG00000204287	HLA-DRA	3,15	4
ENSG00000165949	IFI27	3,01	4

siENSG00000108821	COL1A1	3,00	4
ENSG00000163191	S100A11	2,96	4
ENSG00000087245	MMP2	2,88	4
ENSG00000075624	ACTB	2,76	4
ENSG00000196262	PPIA	2,60	4
ENSG00000231389	HLA-DPA1	2,60	4
ENSG00000165516	KLHDC2	2,59	4
ENSG00000142089	IFITM3	2,53	4
ENSG00000175899	A2M	2,52	4
ENSG00000149131	SERPING1	2,48	4
ENSG00000206503	HLA-A	2,46	4
ENSG00000137965	IFI44	2,41	4
ENSG00000026025	VIM	2,38	4
ENSG00000108679	LGALS3BP	2,38	4
ENSG00000130303	BST2	2,37	4
ENSG00000126709	IFI6	2,35	4
ENSG00000234745	HLA-B	2,32	4
ENSG00000133110	POSTN	2,29	4
ENSG00000129538	RNASE1	2,29	4
ENSG00000113140	SPARC	2,28	4
ENSG00000166710	B2M	2,27	4
ENSG00000142156	COL6A1	2,26	4
ENSG00000135404	CD63	2,21	4
ENSG00000164692	COL1A2	2,16	4
ENSG00000204525	HLA-C	2,16	4
ENSG00000163453	IGFBP7	2,16	4
ENSG00000034510	TMSB10	2,16	4
ENSG00000184009	ACTG1	2,11	4
ENSG00000159403	C1R	1,99	4
ENSG00000123416	TUBA1B	1,97	4
ENSG00000112695	COX7A2	1,87	4
ENSG00000204291	COL15A1	1,86	4
ENSG00000160932	LY6E	1,86	4
ENSG00000135046	ANXA1	1,85	4
ENSG00000187498	COL4A1	1,78	4
ENSG00000185088	RPS27L	1,78	4
ENSG00000163430	FSTL1	1,75	4
ENSG00000107562	CXCL12	1,72	4
ENSG00000115414	FN1	1,70	4
ENSG00000138615	CILP	1,68	4
ENSG00000197747	S100A10	1,62	4
ENSG00000156508	EEF1A1	1,59	4
ENSG00000163513	TGFBR2	1,56	4

ENSG00000159377	PSMB4	1,51	4
ENSG00000134871	COL4A2	1,50	4
ENSG00000168542	COL3A1	1,49	4
ENSG00000101439	CST3	1,43	4
ENSG00000197956	S100A6	1,41	4
ENSG00000163520	FBLN2	1,40	4
ENSG00000112306	RPS12	1,39	4
ENSG00000185559	DLK1	1,38	4
ENSG00000166147	FBN1	1,30	4
ENSG00000086967	MYBPC2	1,30	4
ENSG00000115541	HSPE1	1,11	4
ENSG00000166025	AMOTL1	1,02	4
ENSG00000159251	ACTC1	-1,05	4
ENSG00000140416	TPM1	-1,08	4
ENSG00000154553	PDLIM3	-1,15	4
ENSG00000106211	HSPB1	-1,15	4
ENSG00000129170	CSRP3	-1,26	4
ENSG00000165887	ANKRD2	-1,55	4
ENSG00000101608	MYL12A	-1,65	4
ENSG00000004799	PDK4	-1,73	4
ENSG00000130595	TNNT3	-1,81	4
ENSG00000198712	COX2	-2,07	4
ENSG00000130598	TNNI2	-2,13	4
ENSG00000198899	ATP6	-2,21	4
ENSG00000125414	MYH2	-2,23	4
ENSG00000198786	ND5	-2,32	4
ENSG00000180209	MYL11	-2,38	4
ENSG00000101470	TNNC2	-2,47	4
ENSG00000198727	CYTB	-3,10	4
ENSG00000168530	MYL1	-3,17	4
ENSG00000198804	COX1	-3,21	4
ENSG00000198886	ND4	-3,53	4
ENSG00000198840	ND3	-4,76	4
ENSG00000198938	COX3	-5,09	4
ENSG00000197766	CFD	5,66	5
ENSG00000008517	IL32	3,09	5
ENSG00000148180	GSN	2,96	5
ENSG00000214548	MEG3	2,88	5
ENSG00000158710	TAGLN2	2,70	5
ENSG00000011465	DCN	2,65	5
ENSG00000129170	CSRP3	2,56	5
ENSG00000130598	TNNI2	2,49	5

ENSG00000125414	MYH2	2,38	5
ENSG00000136521	NDUFB5	2,19	5
ENSG00000173641	HSPB7	2,16	5
ENSG00000147684	NDUFB9	2,11	5
ENSG00000130595	TNNT3	2,07	5
ENSG00000163520	FBLN2	2,03	5
ENSG00000101470	TNNC2	1,87	5
ENSG00000135046	ANXA1	1,84	5
ENSG00000163359	COL6A3	1,80	5
ENSG00000159403	C1R	1,74	5
ENSG00000113296	THBS4	1,74	5
ENSG00000204291	COL15A1	1,74	5
ENSG00000108561	C1QBP	1,69	5
ENSG00000163430	FSTL1	1,67	5
ENSG00000117479	SLC19A2	1,64	5
ENSG00000106588	PSMA2	1,61	5
ENSG00000154553	PDLIM3	1,60	5
ENSG00000134871	COL4A2	1,58	5
ENSG00000168542	COL3A1	1,57	5
ENSG00000166147	FBN1	1,52	5
ENSG00000138615	CILP	1,47	5
ENSG00000149131	SERPING1	1,44	5
ENSG00000115541	HSPE1	1,39	5
ENSG00000101439	CST3	1,33	5
ENSG00000178209	PLEC	1,32	5
ENSG00000128591	FLNC	1,32	5
ENSG00000163513	TGFBR2	1,25	5
ENSG00000267855	NDUFA7	1,23	5
ENSG00000197956	S100A6	1,20	5
ENSG00000184076	UQCR10	1,15	5
ENSG00000026025	VIM	1,05	5
ENSG00000155096	AZIN1	1,02	5
ENSG00000198786	ND5	-1,09	5
ENSG00000234745	HLA-B	-1,17	5
ENSG00000198899	ATP6	-1,38	5
ENSG00000198804	COX1	-1,54	5
ENSG00000206503	HLA-A	-1,84	5
ENSG00000198727	CYTB	-2,01	5
ENSG00000198712	COX2	-2,21	5
ENSG00000198886	ND4	-2,73	5

Supplemental Table S2: Indolent and acute JDM muscles display distinct transcriptomic signatures (all results). All significantly enriched Reactome pathways in each signature. Figure 3 provides only the 10 first pathways.

Molecular signature	ID	Description	GeneRatio	BgRatio	pvalue	p.adjust	qvalue	geneID	Count
1	R-HSA-1428517	The citric acid (TCA) cycle and respiratory electron transport	8/8	178/11009	3,99E-15	6,79E-14	4,20E-15	ATP6/ATP8/ND5/ND3/PDK4/COX1/COX3/CYTB	8
1	R-HSA-163200	Respiratory electron transport, ATP synthesis by chemiosmotic coupling, and heat production by uncoupling proteins.	7/8	127/11009	1,82E-13	1,55E-12	9,60E-14	ATP6/ATP8/ND5/ND3/COX1/COX3/CYTB	7
1	R-HSA-611105	Respiratory electron transport	5/8	103/11009	3,56E-09	2,02E-08	1,25E-09	ND5/ND3/COX1/COX3/CYTB	5
1	R-HSA-163210	Formation of ATP by chemiosmotic coupling	2/8	18/11009	7,03E-05	2,99E-04	1,85E-05	ATP6/ATP8	2
1	R-HSA-8949613	Cristae formation	2/8	31/11009	2,13E-04	7,23E-04	4,48E-05	ATP6/ATP8	2
1	R-HSA-6799198	Complex I biogenesis	2/8	57/11009	7,23E-04	2,05E-03	1,27E-04	ND5/ND3	2
1	R-HSA-9707564	Cytoprotection by HMOX1	2/8	64/11009	9,11E-04	2,21E-03	1,37E-04	COX1/COX3	2
1	R-HSA-5628897	TP53 Regulates Metabolic Genes	2/8	87/11009	1,68E-03	3,56E-03	2,21E-04	COX1/COX3	2
1	R-HSA-1592230	Mitochondrial biogenesis	2/8	95/11009	1,99E-03	3,77E-03	2,33E-04	ATP6/ATP8	2
1	R-HSA-9711123	Cellular response to chemical stress	2/8	215/11009	9,84E-03	1,67E-02	1,04E-03	COX1/COX3	2
1	R-HSA-204174	Regulation of pyruvate dehydrogenase (PDH) complex	1/8	16/11009	1,16E-02	1,79E-02	1,11E-03	PDK4	1

1	R-HSA-1852241	Organelle biogenesis and maintenance	2/8	296/11009	1,81E-02	2,57E-02	1,59E-03	ATP6/ATP8	2
1	R-HSA-70268	Pyruvate metabolism	1/8	31/11009	2,23E-02	2,92E-02	1,81E-03	PDK4	1
1	R-HSA-3700989	Transcriptional Regulation by TP53	2/8	362/11009	2,65E-02	3,22E-02	1,99E-03	COX1/COX3	2
1	R-HSA-5362517	Signaling by Retinoic Acid	1/8	43/11009	3,08E-02	3,49E-02	2,16E-03	PDK4	1
1	R-HSA-71406	Pyruvate metabolism and Citric Acid (TCA) cycle	1/8	55/11009	3,93E-02	4,17E-02	2,58E-03	PDK4	1
2	R-HSA-1428517	The citric acid (TCA) cycle and respiratory electron transport	5/8	178/11009	5,63E-08	1,58E-06	4,74E-07	ND4/COX2/CYTB/COX3/PDK4	5
2	R-HSA-611105	Respiratory electron transport	4/8	103/11009	4,92E-07	6,88E-06	2,07E-06	ND4/COX2/CYTB/COX3	4
2	R-HSA-163200	Respiratory electron transport, ATP synthesis by chemiosmotic coupling, and heat production by uncoupling proteins.	4/8	127/11009	1,14E-06	1,06E-05	3,20E-06	ND4/COX2/CYTB/COX3	4
2	R-HSA-9707564	Cytoprotection by HMOX1	3/8	64/11009	1,03E-05	7,20E-05	2,16E-05	HBB/COX2/COX3	3
2	R-HSA-9711123	Cellular response to chemical stress	3/8	215/11009	3,83E-04	2,14E-03	6,44E-04	HBB/COX2/COX3	3
2	R-HSA-5628897	TP53 Regulates Metabolic Genes	2/8	87/11009	1,68E-03	7,82E-03	2,35E-03	COX2/COX3	2
2	R-HSA-1237044	Erythrocytes take up carbon dioxide and release oxygen	1/8	13/11009	9,41E-03	2,93E-02	8,81E-03	HBB	1
2	R-HSA-1480926	O2/CO2 exchange in erythrocytes	1/8	13/11009	9,41E-03	2,93E-02	8,81E-03	HBB	1
2	R-HSA-2168880	Scavenging of heme from plasma	1/8	13/11009	9,41E-03	2,93E-02	8,81E-03	HBB	1

2	R-HSA-204174	Regulation of pyruvate dehydrogenase (PDH) complex	1/8	16/11009	1,16E-02	3,24E-02	9,74E-03	PDK4	1
2	R-HSA-9613829	Chaperone Mediated Autophagy	1/8	22/11009	1,59E-02	4,04E-02	1,22E-02	HBB	1
2	R-HSA-2122948	Activated NOTCH1 Transmits Signal to the Nucleus	1/8	31/11009	2,23E-02	4,81E-02	1,45E-02	DLK1	1
2	R-HSA-70268	Pyruvate metabolism	1/8	31/11009	2,23E-02	4,81E-02	1,45E-02	PDK4	1
2	R-HSA-9615710	Late endosomal microautophagy	1/8	35/11009	2,52E-02	4,94E-02	1,49E-02	HBB	1
2	R-HSA-3700989	Transcriptional Regulation by TP53	2/8	362/11009	2,65E-02	4,94E-02	1,49E-02	COX2/COX3	2
3	R-HSA-397014	Muscle contraction	12/22	205/11009	6,92E-16	4,22E-14	3,20E-14	ACTC1/MYH8/MYH3/MYL12A/SLN/MYL11/MYBPC2/MYL1/CASQ2/TPM1/TNNC2/TNNT3	12
3	R-HSA-390522	Striated Muscle Contraction	8/22	36/11009	1,76E-15	5,36E-14	4,07E-14	ACTC1/MYH8/MYH3/MYBPC2/MYL1/TPM1/TNNC2/TNNT3	8
3	R-HSA-445355	Smooth Muscle Contraction	3/22	45/11009	9,31E-05	1,89E-03	1,44E-03	MYL12A/MYL11/TPM1	3
4	R-HSA-909733	Interferon alpha/beta signaling	12/61	77/11009	7,44E-15	3,27E-12	2,41E-12	ISG15/IFITM1/STAT1/IFIT3/IFIT1/IFI27/IFITM3/HLA-A/BST2/ IFI6 / HLA-B/HLA-C	12
4	R-HSA-913531	Interferon Signaling	16/61	269/11009	7,84E-13	1,72E-10	1,27E-10	ISG15/IFITM1/STAT1/IFIT3/IFIT1/HLA-DRA/IFI27/HLA-DPA1/IFITM3/ HLA-A/BST2/IFI6/HLA-B/B2M/HLA-C/TUBA1B	16
4	R-HSA-1474228	Degradation of the extracellular matrix	11/61	140/11009	2,30E-10	3,37E-08	2,48E-08	COL1A1/MMP2/A2M/COL6A1/COL1A2/COL15A1/COL4A1/FN1/COL4A2/COL3A1/FBN1	11

4	R-HSA-1442490	Collagen degradation	8/61	64/11009	1,92E-09	2,11E-07	1,55E-07	COL1A1/MMP2/COL6A1/COL1A2/COL15A1/COL4A1/COL4A2/COL3A1	8
4	R-HSA-8948216	Collagen chain trimerization	7/61	44/11009	3,67E-09	3,23E-07	2,37E-07	COL1A1/COL6A1/COL1A2/COL15A1/COL4A1/COL4A2/COL3A1	7
4	R-HSA-1474244	Extracellular matrix organization	13/61	300/11009	7,16E-09	4,88E-07	3,59E-07	COL1A1/MMP2/A2M/SPARC/COL6A1/COL1A2/COL15A1/COL4A1/FN1/COL4A2/COL3A1/FBLN2/FBN1	13
4	R-HSA-3000178	ECM proteoglycans	8/61	76/11009	7,77E-09	4,88E-07	3,59E-07	COL1A1/SPARC/COL6A1/COL1A2/COL4A1/FN1/COL4A2/COL3A1	8
4	R-HSA-216083	Integrin cell surface interactions	8/61	85/11009	1,91E-08	1,05E-06	7,70E-07	COL1A1/COL6A1/COL1A2/COL4A1/FN1/COL4A2/COL3A1/FBN1	8
4	R-HSA-2022090	Assembly of collagen fibrils and other multimeric structures	7/61	61/11009	3,89E-08	1,90E-06	1,40E-06	COL1A1/COL6A1/COL1A2/COL15A1/COL4A1/COL4A2/COL3A1	7
4	R-HSA-3000480	Scavenging by Class A Receptors	5/61	19/11009	4,84E-08	2,13E-06	1,56E-06	COL1A1/COL1A2/COL4A1/COL4A2/COL3A1	5
4	R-HSA-1650814	Collagen biosynthesis and modifying enzymes	7/61	67/11009	7,55E-08	3,02E-06	2,22E-06	COL1A1/COL6A1/COL1A2/COL15A1/COL4A1/COL4A2/COL3A1	7
4	R-HSA-2173782	Binding and Uptake of Ligands by Scavenger Receptors	6/61	42/11009	1,01E-07	3,71E-06	2,72E-06	COL1A1/SPARC/COL1A2/COL4A1/COL4A2/COL3A1	6
4	R-HSA-1236977	Endosomal/Vacuolar pathway	4/61	11/11009	2,73E-07	9,26E-06	6,80E-06	HLA-A/HLA-B/B2M/HLA-C	4
4	R-HSA-1474290	Collagen formation	7/61	90/11009	5,87E-07	1,85E-05	1,36E-05	COL1A1/COL6A1/COL1A2/COL15A1/COL4A1/COL4A2/COL3A1	7
4	R-HSA-198933	Immunoregulatory interactions between a Lymphoid and a non-Lymphoid cell	8/61	133/11009	6,32E-07	1,85E-05	1,36E-05	IFITM1/COL1A1/HLA-A/HLA-B/B2M/COL1A2/HLA-C/COL3A1	8

4	R-HSA-3000171	Non-integrin membrane-ECM interactions	6/61	59/11009	8,07E-07	2,22E-05	1,63E-05	COL1A1/COL1A2/COL4A1/FN1/COL4A2/COL3A1	6
4	R-HSA-877300	Interferon gamma signaling	7/61	96/11009	9,13E-07	2,36E-05	1,74E-05	STAT1/HLA-DRA/HLA-DPA1/HLA-A/ HLA-B/B2M/HLA-C	7
4	R-HSA-2214320	Anchoring fibril formation	4/61	15/11009	1,11E-06	2,72E-05	2,00E-05	COL1A1/COL1A2/COL4A1/COL4A2	4
4	R-HSA-2243919	Crosslinking of collagen fibrils	4/61	18/11009	2,46E-06	5,70E-05	4,19E-05	COL1A1/COL1A2/COL4A1/COL4A2	4
4	R-HSA-114608	Platelet degranulation	7/61	129/11009	6,64E-06	1,46E-04	1,07E-04	PPIA/A2M/SERPING1/LGALS3BP/SPARC/CD63/FN1	7
4	R-HSA-76005	Response to elevated platelet cytosolic Ca2+	7/61	134/11009	8,54E-06	1,79E-04	1,31E-04	PPIA/A2M/SERPING1/LGALS3BP/SPARC/CD63/FN1	7
4	R-HSA-76002	Platelet activation, signaling and aggregation	9/61	263/11009	1,29E-05	2,59E-04	1,90E-04	COL1A1/PPIA/A2M/SERPING1/LGALS3BP/SPARC/CD63/COL1A2/FN1	9
4	R-HSA-3000170	Syndecan interactions	4/61	27/11009	1,36E-05	2,60E-04	1,91E-04	COL1A1/COL1A2/FN1/COL3A1	4
4	R-HSA-9679506	SARS-CoV Infections	11/61	414/11009	1,43E-05	2,61E-04	1,92E-04	ISG15/STAT1/PPIA/HLA-A/BST2/HLA-B/B2M/HLA-C/RPS27L/EEF1A1/RPS12	11
4	R-HSA-9705683	SARS-CoV-2-host interactions	8/61	203/11009	1,48E-05	2,61E-04	1,92E-04	ISG15/STAT1/HLA-A/HLA-B/B2M/HLA-C/RPS27L/RPS12	8
4	R-HSA-186797	Signaling by PDGF	5/61	58/11009	1,62E-05	2,74E-04	2,01E-04	STAT1/COL6A1/COL4A1/COL4A2/COL3A1	5
4	R-HSA-983170	Antigen Presentation: Folding, assembly and peptide loading of class I MHC	4/61	29/11009	1,83E-05	2,98E-04	2,19E-04	HLA-A/HLA-B/B2M/HLA-C	4
4	R-HSA-8874081	MET activates PTK2 signaling	4/61	30/11009	2,10E-05	3,30E-04	2,42E-04	COL1A1/COL1A2/FN1/COL3A1	4
4	R-HSA-6785807	Interleukin-4 and Interleukin-13 signaling	6/61	108/11009	2,78E-05	4,21E-04	3,10E-04	STAT1/MMP2/VIM/COL1A2/ANXA1/FN1	6

4	R-HSA-166786	Creation of C4 and C2 activators	3/61	14/11009	5,64E-05	8,27E-04	6,08E-04	C1QB/C1S/C1R	3
4	R-HSA-381426	Regulation of Insulin-like Growth Factor (IGF) transport and uptake by Insulin-like Growth Factor Binding Proteins (IGFBPs)	6/61	125/11009	6,33E-05	8,99E-04	6,60E-04	MMP2/IGFBP7/FSTL1/FN1/CST3/FBN1	6
4	R-HSA-9705671	SARS-CoV-2 activates/modulates innate and adaptive immune responses	6/61	126/11009	6,62E-05	9,10E-04	6,68E-04	ISG15/STAT1/HLA-A/HLA-B/B2M/HLA-C	6
4	R-HSA-8875878	MET promotes cell motility	4/61	41/11009	7,41E-05	9,88E-04	7,26E-04	COL1A1/COL1A2/FN1/COL3A1	4
4	R-HSA-419037	NCAM1 interactions	4/61	42/11009	8,16E-05	1,06E-03	7,75E-04	COL6A1/COL4A1/COL4A2/COL3A1	4
4	R-HSA-977606	Regulation of Complement cascade	4/61	47/11009	1,27E-04	1,60E-03	1,18E-03	C1QB/C1S/SERPING1/C1R	4
4	R-HSA-1236974	ER-Phagosome pathway	5/61	90/11009	1,35E-04	1,66E-03	1,22E-03	HLA-A/HLA-B/B2M/HLA-C/PSMB4	5
4	R-HSA-9692914	SARS-CoV-1-host interactions	5/61	96/11009	1,84E-04	2,18E-03	1,60E-03	PPIA/BST2/RPS27L/EEF1A1/RPS12	5
4	R-HSA-9694516	SARS-CoV-2 Infection	8/61	299/11009	2,27E-04	2,61E-03	1,92E-03	ISG15/STAT1/HLA-A/HLA-B/B2M/HLA-C/RPS27L/RPS12	8
4	R-HSA-9613829	Chaperone Mediated Autophagy	3/61	22/11009	2,31E-04	2,61E-03	1,92E-03	VIM/RNASE1/EEF1A1	3
4	R-HSA-166663	Initial triggering of complement	3/61	23/11009	2,65E-04	2,91E-03	2,14E-03	C1QB/C1S/C1R	3
4	R-HSA-1236975	Antigen processing-Cross presentation	5/61	105/11009	2,79E-04	3,00E-03	2,20E-03	HLA-A/HLA-B/B2M/HLA-C/PSMB4	5
4	R-HSA-166658	Complement cascade	4/61	58/11009	2,89E-04	3,03E-03	2,23E-03	C1QB/C1S/SERPING1/C1R	4

4	R-HSA-8957275	Post-translational protein phosphorylation	5/61	108/11009	3,18E-04	3,26E-03	2,39E-03	IGFBP7/FSTL1/FN1/CST3/FBN1	5
4	R-HSA-375165	NCAM signaling for neurite out-growth	4/61	63/11009	3,98E-04	3,98E-03	2,92E-03	COL6A1/COL4A1/COL4A2/COL3A1	4
4	R-HSA-2132295	MHC class II antigen presentation	5/61	123/11009	5,79E-04	5,66E-03	4,16E-03	LGMN/CD74/HLA-DRA/HLA-DPA1/TUBA1B	5
4	R-HSA-9006936	Signaling by TGFB family members	5/61	124/11009	6,01E-04	5,75E-03	4,22E-03	STAT1/COL1A2/FSTL1/TGFBR2/FBN1	5
4	R-HSA-5626467	RHO GTPases activate IQGAPs	3/61	32/11009	7,16E-04	6,70E-03	4,92E-03	ACTB/ACTG1/TUBA1B	3
4	R-HSA-6806834	Signaling by MET	4/61	79/11009	9,40E-04	8,47E-03	6,22E-03	COL1A1/COL1A2/FN1/COL3A1	4
4	R-HSA-202733	Cell surface interactions at the vascular wall	5/61	137/11009	9,44E-04	8,47E-03	6,22E-03	CD74/COL1A1/PPIA/COL1A2/FN1	5
4	R-HSA-6802948	Signaling by high-kinase activity BRAF mutants	3/61	36/11009	1,01E-03	8,93E-03	6,56E-03	ACTB/ACTG1/FN1	3
4	R-HSA-2129379	Molecules associated with elastic fibres	3/61	37/11009	1,10E-03	9,20E-03	6,76E-03	FN1/FBLN2/FBN1	3
4	R-HSA-9735869	SARS-CoV-1 modulates host translation machinery	3/61	37/11009	1,10E-03	9,20E-03	6,76E-03	RPS27L/EEF1A1/RPS12	3
4	R-HSA-9678108	SARS-CoV-1 Infection	5/61	142/11009	1,11E-03	9,20E-03	6,76E-03	PPIA/BST2/RPS27L/EEF1A1/RPS12	5
4	R-HSA-6798695	Neutrophil degranulation	9/61	480/11009	1,21E-03	9,89E-03	7,27E-03	S100A11/PPIA/BST2/HLA-B/B2M/CD63/HLA-C/EEF1A1/CST3	9
4	R-HSA-76009	Platelet Aggregation (Plug Formation)	3/61	39/11009	1,28E-03	1,03E-02	7,54E-03	COL1A1/COL1A2/FN1	3
4	R-HSA-164940	Nef mediated downregulation of MHC class I complex cell surface expression	2/61	10/11009	1,32E-03	1,04E-02	7,62E-03	HLA-A/B2M	2

4	R-HSA-5674135	MAP2K and MAPK activation	3/61	40/11009	1,38E-03	1,07E-02	7,84E-03	ACTB/ACTG1/FN1	3
4	R-HSA-196025	Formation of annular gap junctions	2/61	11/11009	1,61E-03	1,22E-02	8,96E-03	ACTB/ACTG1	2
4	R-HSA-9656223	Signaling by RAF1 mutants	3/61	43/11009	1,71E-03	1,27E-02	9,35E-03	ACTB/ACTG1/FN1	3
4	R-HSA-170834	Signaling by TGF-beta Receptor Complex	4/61	94/11009	1,79E-03	1,32E-02	9,66E-03	STAT1/COL1A2/TGFBR2/FBN1	4
4	R-HSA-1566948	Elastic fibre formation	3/61	44/11009	1,82E-03	1,32E-02	9,66E-03	FN1/FBLN2/FBN1	3
4	R-HSA-190873	Gap junction degradation	2/61	12/11009	1,92E-03	1,34E-02	9,83E-03	ACTB/ACTG1	2
4	R-HSA-430116	GP1b-IX-V activation signalling	2/61	12/11009	1,92E-03	1,34E-02	9,83E-03	COL1A1/COL1A2	2
4	R-HSA-2172127	DAP12 interactions	3/61	45/11009	1,95E-03	1,34E-02	9,83E-03	HLA-B/B2M/HLA-C	3
4	R-HSA-190828	Gap junction trafficking	3/61	47/11009	2,21E-03	1,39E-02	1,02E-02	ACTB/ACTG1/TUBA1B	3
4	R-HSA-437239	Recycling pathway of L1	3/61	47/11009	2,21E-03	1,39E-02	1,02E-02	ACTB/ACTG1/TUBA1B	3
4	R-HSA-6802946	Signaling by moderate kinase activity BRAF mutants	3/61	47/11009	2,21E-03	1,39E-02	1,02E-02	ACTB/ACTG1/FN1	3
4	R-HSA-6802949	Signaling by RAS mutants	3/61	47/11009	2,21E-03	1,39E-02	1,02E-02	ACTB/ACTG1/FN1	3
4	R-HSA-6802955	Paradoxical activation of RAF signaling by kinase inactive BRAF	3/61	47/11009	2,21E-03	1,39E-02	1,02E-02	ACTB/ACTG1/FN1	3
4	R-HSA-9649948	Signaling downstream of RAS mutants	3/61	47/11009	2,21E-03	1,39E-02	1,02E-02	ACTB/ACTG1/FN1	3
4	R-HSA-157858	Gap junction trafficking and regulation	3/61	49/11009	2,49E-03	1,54E-02	1,13E-02	ACTB/ACTG1/TUBA1B	3

4	R-HSA-3928665	EPH-ephrin mediated repulsion of cells	3/61	51/11009	2,79E-03	1,71E-02	1,25E-02	MMP2/ACTB/ACTG1	3
4	R-HSA-75892	Platelet Adhesion to exposed collagen	2/61	15/11009	3,03E-03	1,82E-02	1,34E-02	COL1A1/COL1A2	2
4	R-HSA-449147	Signaling by Interleukins	8/61	473/11009	4,35E-03	2,56E-02	1,88E-02	STAT1/MMP2/PPIA/VIM/COL1A2/ANXA1/FN1/PSMB4	8
4	R-HSA-446353	Cell-extracellular matrix interactions	2/61	18/11009	4,36E-03	2,56E-02	1,88E-02	ACTB/ACTG1	2
4	R-HSA-202430	Translocation of ZAP-70 to Immunological synapse	2/61	19/11009	4,86E-03	2,81E-02	2,07E-02	HLA-DRA/HLA-DPA1	2
4	R-HSA-162909	Host Interactions of HIV factors	4/61	130/11009	5,76E-03	3,29E-02	2,42E-02	PPIA/HLA-A/B2M/PSMB4	4
4	R-HSA-164938	Nef-mediates down modulation of cell surface receptors by recruiting them to clathrin adapters	2/61	21/11009	5,93E-03	3,34E-02	2,46E-02	HLA-A/B2M	2
4	R-HSA-6802952	Signaling by BRAF and RAF1 fusions	3/61	67/11009	6,03E-03	3,36E-02	2,47E-02	ACTB/ACTG1/FN1	3
4	R-HSA-202427	Phosphorylation of CD3 and TCR zeta chains	3/61	22/11009	6,50E-03	3,57E-02	2,62E-02	HLA-DRA/HLA-DPA1	2
4	R-HSA-140837	Intrinsic Pathway of Fibrin Clot Formation	2/61	23/11009	7,09E-03	3,80E-02	2,79E-02	A2M/SERPING1	2
4	R-HSA-389948	PD-1 signaling	3/61	23/11009	7,09E-03	3,80E-02	2,79E-02	HLA-DRA/HLA-DPA1	2
4	R-HSA-1445148	Translocation of SLC2A4 (GLUT4) to the plasma membrane	3/61	72/11009	7,36E-03	3,90E-02	2,87E-02	ACTB/ACTG1/TUBA1B	3
4	R-HSA-1169408	ISG15 antiviral mechanism	3/61	73/11009	7,65E-03	4,00E-02	2,94E-02	ISG15/STAT1/IFIT1	3
4	R-HSA-9833482	PKR-mediated signaling	3/61	75/11009	8,24E-03	4,26E-02	3,13E-02	ISG15/STAT1/TUBA1B	3

4	R-HSA-1169410	Antiviral mechanism by IFN-stimulated genes	4/61	149/11009	9,26E-03	4,74E-02	3,48E-02	ISG15/STAT1/IFIT1/TUBA1B	4
4	R-HSA-9612973	Autophagy	4/61	151/11009	9,69E-03	4,90E-02	3,60E-02	VIM/RNASE1/TUBA1B/EEF1A1	4
5	R-HSA-2022090	Assembly of collagen fibrils and other multimeric structures	5/35	61/11009	1,26E-06	2,83E-04	2,32E-04	COL6A3/COL15A1/COL4A2/COL3A1/PLEC	5
5	R-HSA-1474244	Extracellular matrix organization	8/35	300/11009	3,43E-06	2,83E-04	2,32E-04	DCN/FBLN2/COL6A3/COL15A1/COL4A2/COL3A1/FBN1/PLEC	8
5	R-HSA-1474228	Degradation of the extracellular matrix	6/35	140/11009	4,55E-06	2,83E-04	2,32E-04	DCN/COL6A3/COL15A1/COL4A2/COL3A1/FBN1	6
5	R-HSA-390522	Striated Muscle Contraction	4/35	36/11009	4,69E-06	2,83E-04	2,32E-04	TNNI2/TNNT3/TNNC2/VIM	4
5	R-HSA-264870	Caspase-mediated cleavage of cytoskeletal proteins	3/35	12/11009	6,35E-06	3,06E-04	2,51E-04	GSN/PLEC/VIM	3
5	R-HSA-1474290	Collagen formation	5/35	90/11009	8,73E-06	3,51E-04	2,88E-04	COL6A3/COL15A1/COL4A2/COL3A1/PLEC	5
5	R-HSA-8948216	Collagen chain trimerization	4/35	44/11009	1,06E-05	3,66E-04	3,00E-04	COL6A3/COL15A1/COL4A2/COL3A1	4
5	R-HSA-186797	Signaling by PDGF	4/35	58/11009	3,22E-05	9,69E-04	7,95E-04	COL6A3/THBS4/COL4A2/COL3A1	4
5	R-HSA-1442490	Collagen degradation	4/35	64/11009	4,75E-05	1,27E-03	1,04E-03	COL6A3/COL15A1/COL4A2/COL3A1	4
5	R-HSA-1650814	Collagen biosynthesis and modifying enzymes	4/35	67/11009	5,69E-05	1,37E-03	1,13E-03	COL6A3/COL15A1/COL4A2/COL3A1	4
5	R-HSA-3000178	ECM proteoglycans	4/35	76/11009	9,34E-05	2,05E-03	1,68E-03	DCN/COL6A3/COL4A2/COL3A1	4
5	R-HSA-216083	Integrin cell surface interactions	4/35	85/11009	1,44E-04	2,90E-03	2,38E-03	COL6A3/COL4A2/COL3A1/FBN1	4
5	R-HSA-111465	Apoptotic cleavage of cellular proteins	3/35	38/11009	2,30E-04	4,15E-03	3,41E-03	GSN/PLEC/VIM	3
5	R-HSA-109581	Apoptosis	5/35	180/11009	2,41E-04	4,15E-03	3,41E-03	GSN/C1QBP/PSMA2/PLEC/VIM	5

5	R-HSA-611105	Respiratory electron transport	4/35	103/11009	3,03E-04	4,68E-03	3,84E-03	NDUFB5/NDUFB9/NDUFA7/UQCR10	4
5	R-HSA-419037	NCAM1 interactions	3/35	42/11009	3,10E-04	4,68E-03	3,84E-03	COL6A3/COL4A2/COL3A1	3
5	R-HSA-397014	Muscle contraction	5/35	205/11009	4,39E-04	6,23E-03	5,11E-03	TNNI2/TNNT3/TNNC2/ANXA1/VIM	5
5	R-HSA-5357801	Programmed Cell Death	5/35	213/11009	5,23E-04	7,01E-03	5,75E-03	GSN/C1QBP/PSMA2/PLEC/VIM	5
5	R-HSA-75153	Apoptotic execution phase	3/35	52/11009	5,85E-04	7,42E-03	6,09E-03	GSN/PLEC/VIM	3
5	R-HSA-163200	Respiratory electron transport, ATP synthesis by chemiosmotic coupling, and heat production by uncoupling proteins.	4/35	127/11009	6,70E-04	8,08E-03	6,63E-03	NDUFB5/NDUFB9/NDUFA7/UQCR10	4
5	R-HSA-6799198	Complex I biogenesis	3/35	57/11009	7,66E-04	8,79E-03	7,22E-03	NDUFB5/NDUFB9/NDUFA7	3
5	R-HSA-166658	Complement cascade	3/35	58/11009	8,06E-04	8,83E-03	7,25E-03	CFD/C1R/SERPING1	3
5	R-HSA-375165	NCAM signaling for neurite out-growth	3/35	63/11009	1,03E-03	1,07E-02	8,83E-03	COL6A3/COL4A2/COL3A1	3
5	R-HSA-3000480	Scavenging by Class A Receptors	2/35	19/11009	1,62E-03	1,63E-02	1,34E-02	COL4A2/COL3A1	2
5	R-HSA-1428517	The citric acid (TCA) cycle and respiratory electron transport	4/35	178/11009	2,34E-03	2,13E-02	1,75E-02	NDUFB5/NDUFB9/NDUFA7/UQCR10	4
5	R-HSA-140837	Intrinsic Pathway of Fibrin Clot Formation	2/35	23/11009	2,38E-03	2,13E-02	1,75E-02	C1QBP/SERPING1	2
5	R-HSA-166663	Initial triggering of complement	2/35	23/11009	2,38E-03	2,13E-02	1,75E-02	CFD/C1R	2
5	R-HSA-8957275	Post-translational protein phosphorylation	3/35	108/11009	4,78E-03	4,12E-02	3,38E-02	FSTL1/FBN1/CST3	3

Supplemental Figure S1.

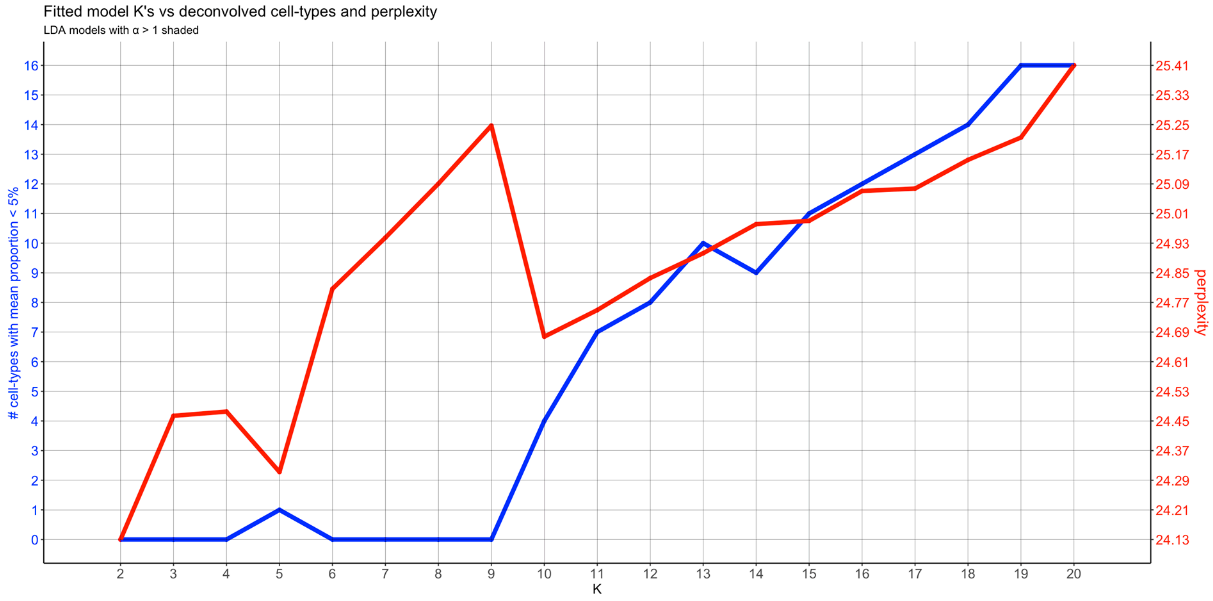

Selection of appropriate number of transcriptomics signatures K a priori. In general, the model perplexity (red curve) is decreasing depending on K, but an increasing perplexity is not problematic if the LDA shape parameter $\alpha < 1$. The optimal K should theoretically minimize the perplexity in accordance with the number of rare transcriptomics signatures that could be unveiled (blue curve). After K=2 (irrelevant), the best models is found for K=5. See Stdeconvolve publication Miller et al. (2022) for more details.

Supplemental Figure S2.

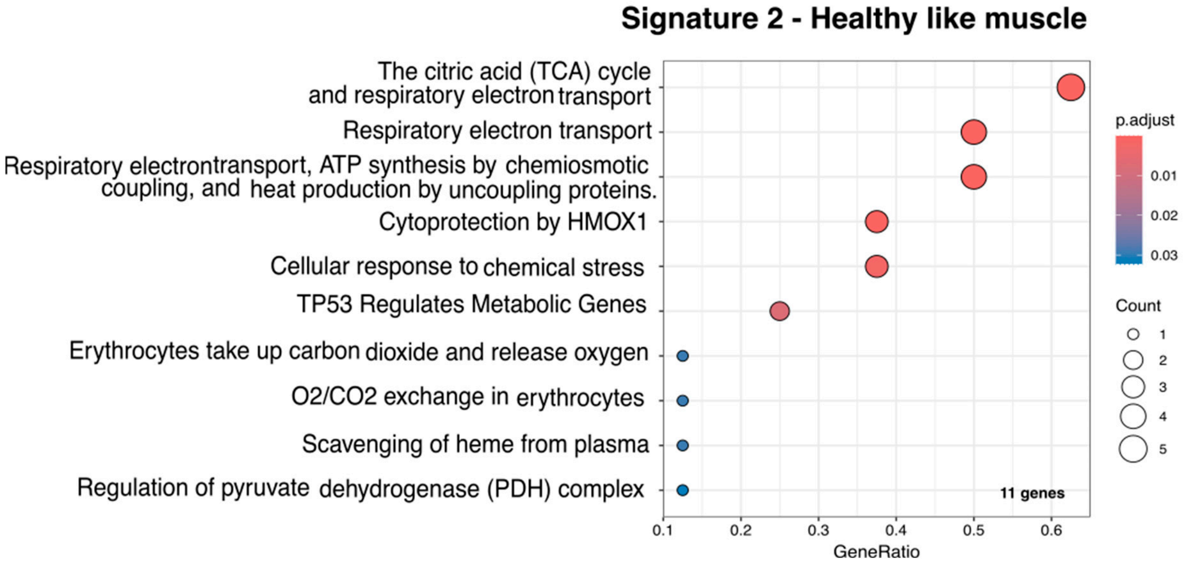

Ten first enriched Reactome pathways. Queries were the up regulated genes from marker genes list (log2fc > 1) for signature 2 unveiled by de novo deconvolution.
